# Prolactin Promotes Breast Cancer Cell Migration through Actin Cytoskeleton Remodeling

**DOI:** 10.3389/fendo.2015.00186

**Published:** 2015-12-17

**Authors:** Priscilla Ludovico da Silva, Vinicius Cestari do Amaral, Valentina Gabrielli, Maria Magdalena Montt Guevara, Paolo Mannella, Edmund Chada Baracat, Jose Maria Soares-Jr, Tommaso Simoncini

**Affiliations:** ^1^Department of Clinical and Experimental Medicine, University of Pisa, Pisa, Italy; ^2^Laboratory of Structural and Molecular Gynecology (LIM58), Discipline of Gynecology, Department of Obstetrics and Gynecology, University of São Paulo, São Paulo, Brazil; ^3^Health Sciences Institute (ICS), Paulista University, São Paulo, Brazil

**Keywords:** T47D, MCF-7, ZR75-1, breast cancer, prolactin, cell migration, actin cytoskeleton

## Abstract

The role of prolactin on breast cancer development and progression is debated. Breast cancer progression largely depends on cell movement and on the ability to remodel the actin cytoskeleton. In this process, actin-binding proteins are requested to achieve fibrillar actin de-polymerization and relocation at the cell membrane. Kinases such as focal adhesion kinase (FAK) are later required to form actin/vinculin-enriched structures called focal adhesion complexes, which mediate firm adhesion to the extracellular matrix. These controllers are regulated by c-Src, which forms multiprotein signaling complexes with membrane receptors and is regulated by a number of hormones, including ­prolactin. We here show that breast cancer cells exposed to prolactin display an elevated c-Src expression and phosphorylation. In parallel, increased moesin and FAK expression and phosphorylation are found. These molecular changes are associated to relocation to the plasma membrane of cytoskeletal actin fibers and to increased horizontal cell movement. In conclusion, prolactin regulates actin remodeling and enhances breast cancer cell movement. This finding broadens the understanding of prolactin actions on breast cancer cells, highlighting new pathways that may be relevant to on breast cancer progression.

## Introduction

In the recent past, prolactin (PRL) has been recognized to have broader actions than previously thought. While first identified as the key hormone mediating growth and differentiation of mammary epithelium and lactation, PRL has recently gained attention for its role in the development of breast cancer (1, 2). PRL effects on breast cancer are mediated by interaction with its receptor (PRLR), a member of the cytokine receptor superfamily, characterized by a tripartite structure: an extracellular ligand-binding domain, a short transmembrane domain, and an intracellular domain. PRLR signaling is triggered by the contemporary interaction of PRL with two receptors: a high affinity PRL site binds one receptor, and a lower affinity PRL site binds another receptor, thus favoring the formation of a ternary complex comprised of PRL and two PRLR (3).

High PRL levels are directly linked with breast cancer risk (4) and with worse prognosis in breast cancer patients (5). Transgenic rodents overexpressing PRLR in the breast develop mammary tumors with a high frequency (6). PRL also antagonizes cytotoxicity by chemotherapeutic agents, thus reducing the efficacy of available treatments (2).

Prolactin receptor is found in up to 80% of breast cancer cells (7), where it couples to several signaling pathways, including Janus Kinase (JAK)-2/Signal Transducer and Activator of Transcription (STAT)-5, mitogen-activated protein kinase (MAPK), and phosphatidylinositol-3-OH-kinase (PI3K). Activation of these pathways directly impacts proliferation, survival, and cytoskeletal dynamics, thus influencing initiation and progression of mammary tumors (1). On these grounds, PRLR is currently being exploited as a potential therapeutic target in breast cancer (3).

Activation of MAPK and PI3K is an established mechanism through which sex steroid hormones modulate the architecture of the cytoskeleton in breast cancer cells (8). The actin cytoskeleton forms the backbone of the cell, and its spatial organization is crucial for cell movement. Modification of the intracellular localization of actin fibers and of their interaction with membrane-anchoring structures, such as integrins and focal adhesion complexes, allows cell movement in the extracellular environment and is directly linked to the ability to achieve local and distant metastasis (9).

Prolactin is able to recruit signaling intermediates such as c-Src that are upstream controllers of PI3K and focal adhesion kinase (FAK) in breast cancer cells (10). Activation of PI3K by PRL results from either direct binding to PRLR or from Src activation (1). This is remarkably similar to the regulatory actions of estrogen receptor alpha (ER alpha) (11), that is, an established modulator of the actin cytoskeleton in breast cancer cells through the recruitment of c-Src-mediated signaling to actin-regulatory proteins and FAK (8).

It is currently unclear whether PRL/PRLR signaling may turn into modifications that have an impact on breast cancer cell movement and possibly metastasis through the control of actin cytoskeleton rearrangement. The aim of this work has been to explore this possibility, through the analysis of breast cancer cell horizontal movement during exposure to PRL, through the study of the cytoskeletal modifications induced in these cells by PRL, and by a preliminary characterization of the molecular pathways involved.

## Materials and Methods

### Cell Culture and Treatments

T47D (ATCC^®^HTB-133™), ZR75-1 (ATCC^®^CRL-1500™), and MCF-7 (ATCC^®^HTB-22™) human breast cancer cells were obtained from the American Tissue Culture Collection (ATCC^®^, Manassas, VA, USA) and cultured as previously described (12). T47D and ZR75-1 cells were maintained in Roswell Park Memorial Institute 1640 Medium (1X) (RPMI). MCF-7 cells were maintained in Dulbecco’s Modified Eagles’s Medium (DMEM). The culture mediums were supplemented with 10% of fetal bovine serum (FBS), 1.2% of l-glutamine, 1.2% of penicillin, 1.2% of [4-(2-hydroxyethyl)piperazin-1-yl]ethanesulfonic acid (HEPES), 1.2% of pyruvate, 2.4% of sodium bicarbonate, and 0.24% of insulin. The cells were plated in different dishes (six well, 35 mm diameter) at a density of 2 × 10^4^ cells/ml (2 ml of RPMI or DMEM), at a 37°C in 5% CO_2_. The mediums and the FBS were purchased from Gibco (Grand Island, NY, USA). All others reagents were purchased from Sigma-Aldrich (Gillingham, England). Before treatments, the cells were kept for 48 h in culture medium containing FBS deprived of steroids with charcoal stripping. Before experiments, the cells were kept for 8 h in culture medium containing no FBS. We treated the cells with three different doses of PRL: 25, 50, and 100 ng/ml, as previously described (10). Prolactin was purchased from Abnova (P4122, Human Recombinant Protein, Taiwan).

### Gene Silencing with siRNA and Transfection Experiment

To transfect anti-hPRLRsiRNA (AM16708-ID106337, ambiom^®^, ThermoFisher Scientific, Grand Island, NY, USA), cells were plated at 70% confluence in six-well dishes. After 12 h, cells were transfected with siRNA using Lipofectamine 2000 Transfection Reagent (ThermoFisher Scientific, Grand Island, NY, USA) according to the manufacturer’s instructions. Briefly, siRNA was diluted at a concentration of 30 nM in culture medium, in a final volume of 100 μl, mixed by vortexing, and then 6 μl lipofectamine was added to the diluted siRNA. The samples were mixed by pipetting and incubated for 20 min at room temperature. Then, the transfection complex was added to 2 ml medium without antibiotics. After 6 h, when the transfection occurred, new cell growth medium containing serum and antibiotics was added to the cells. Cells were incubated with the transfection complex under their normal growth conditions and monitored for gene silencing after 48 h (13).

### Cell-Migration Assays

Cell migration was assayed with razor-scrape assays as previously described (14). Briefly, a razor blade was pressed trough the confluent T47D, ZR75-1, and MCF-7 monolayer into the plastic plate to mark the starting line. T47D, ZR75-1, and MCF-7 were swept away on one side of that line. Cells were washed, and 2.0 ml of RPMI (T47-D and ZR75-1) and DMEM (MCF-7) containing steroid-deprived FBS were added. Cytosine β-d-arabinofuranoside hydrochloride (Sigma-Aldrich, Gillingham, England) (10 μM) was used 1 h before the hormone was added. Migration was monitored for 48 h (15, 16). Every 12 h, fresh medium and treatment were replaced. Cells were digitally imaged and migration distance in micrometer was measured with phase-contrast microscopy (Axiolab Standart 2.0 Carl Zeiss, Jena, Germany).

### Cell Immunofluorescence

After exposure to 30 min of treatment with PRL, cells were fixed with 4% paraformaldehyde for 30 min and permeabilized with 0.1% Triton for 5 min. Blocking was performed with 3% normal serum for 30 min. Cells were incubated with Texas red-linked antibody against Phalloidin (Sigma-Aldrich, Gillingham, England). The nuclei were counterstained with 4′,6-diamidino-2-phenylindole (DAPI) (Sigma-Aldrich, Gillingham, England). Immunofluorescence was visualized using an Olympus BX41 microscope and recorded with a high-resolution DP70 Olympus digital camera. After conversion to gray scale images, the cell membrane thickness and the gray levels of the extracellular area, cell membrane, as well as cytoplasm were quantified, as previously described (17) using the LeicaQWin image analysis and image processing software (Leica Microsystems, Wetzlar, Germany). We analyzed, in 50 cells per condition, an area of 40 pixel-distance encompassing the extracellular space, the full thickness of the membrane and the intracellular space. Five separate measures were taken in each cell.

### Immunoblottings

Cell lysates were separated by sodium dodecyl sulphate (SDS) polyacrylamide gel electrophoresis (PAGE). Primary antibodies used were: PRLR (D01PID5618, Abnova, Taiwan); Moesin (SC6410, Santa Cruz, Texas, USA); p-Moesin (SC12895, Santa Cruz, Texas, USA); FAK (SC271195, Santa Cruz, Texas, USA); p-FAK (SC11765-R, Santa Cruz, Texas, USA); c-Src (SC5266, Santa Cruz, Texas, USA); p-c-Src (SC166860, Santa Cruz, Texas, USA); GAPDH (SC59540, Santa Cruz, Texas, USA). The secondary antibodies Anti-rabbit (SC2357, Santa Cruz, Texas, USA) (1:3000) for PRLR; Anti-goat (SC2768, Santa Cruz, Texas, USA) (1:3000) for Moesin, p-Moesin and Actin, and Anti-Mouse (SC2005, Santa Cruz, Texas, USA (1:3500) for FAK, p-FAK, c-Src, p-c-Src, and GAPDH were incubated for 2 h in room temperature. Primary and secondary antibodies were incubated with the membranes, followed by three 5-min washings with TRIS-buffered saline-Tween 20. Immunodetection was carried out using enhanced chemiluminescence and was recorded with a quantitative digital imaging system (Quantity One, BioRad, Hercules, CA, USA), enabling us to assess saturation. Band densitometric analysis was quantified, as previously described (17), with the ImageJ image program (National Institute of Health – NIH, USA) using conditions ensuring analysis in the linear range of detection.

### Statistical Analyses

The statistical analysis was performed using the software GrahPad Prism 6.03 (GraphPad Software Inc., CA, USA). The data were analyzed using a Kruskal–Wallis test followed by Dunn’s *post hoc* test. *p* < 0.05 was considered to be significant.

The results were expressed as mean ± SD.

## Results

### PRL Enhances T47D, ZR75-1, and MCF-7 Cell Migration

**Figure 1:**
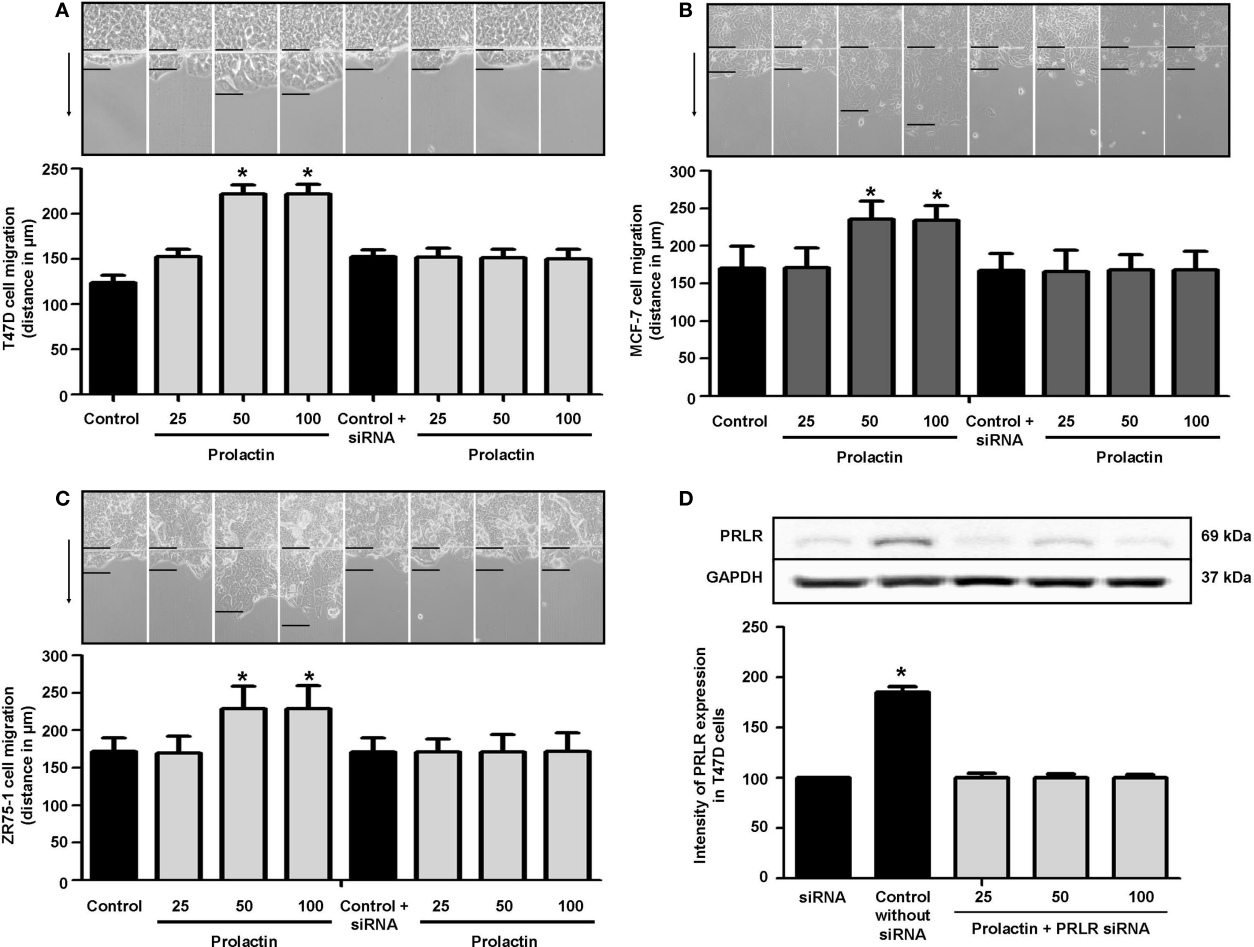
**(A)** Migration of T47D cells 48 h after administration of different PRL doses (nanogram per milliliter) with or without anti-hPRLR siRNA. Images were converted into a spectrum of grays. The arrow points to the direction of migration. The upper black line is where migration begins, and the lower black line shows the maximal migration distance. The distance covered by the migrating cells was measured in micrometers. *p < 0.05 vs. control. The representative images show T47D **(A)**, MCF-7 **(B)**, and ZR75-1 **(C)** migration. Each experiment was repeated three times. **(D)** shows PRLR expression after different PRL doses (nanogram per milliliter) in the presence or absence of anti-hPRLR siRNA. PRLR densitometry value was adjusted for GAPDH and then normalized to control (Con). The experiment was repeated four times. *p < 0.05 versus control.

To assess whether PRL influences breast cancer cell motility, we pretreated T47D, ZR75-1, and MCF-7 cells with cytosine β-d-arabinofuranoside hydrochloride, a selective inhibitor of DNA synthesis that does not inhibit RNA synthesis, and we performed horizontal migration assays as previously described (15, 16). The PRL doses of 50 and 100 ng/ml significantly increased mean length (micrometer) of migration after 48 h of treatment (Figure 1A) in T47D cells (Figure 1A), MCF-7 cells (Figure 1B), and ZR75-1 cells (Figure 1C). PRL acted through PRLR, as shown by the addition of PRLR-siRNAs that results in effective silencing of PRLR expression (Figure 1D). In this condition, no increase in migration is seen in the presence of PRL (Figures 1A–C). These data were similar for all cell lines of this study.

### PRL Induces Actin Cytoskeleton Remodeling

**Figure 2:**
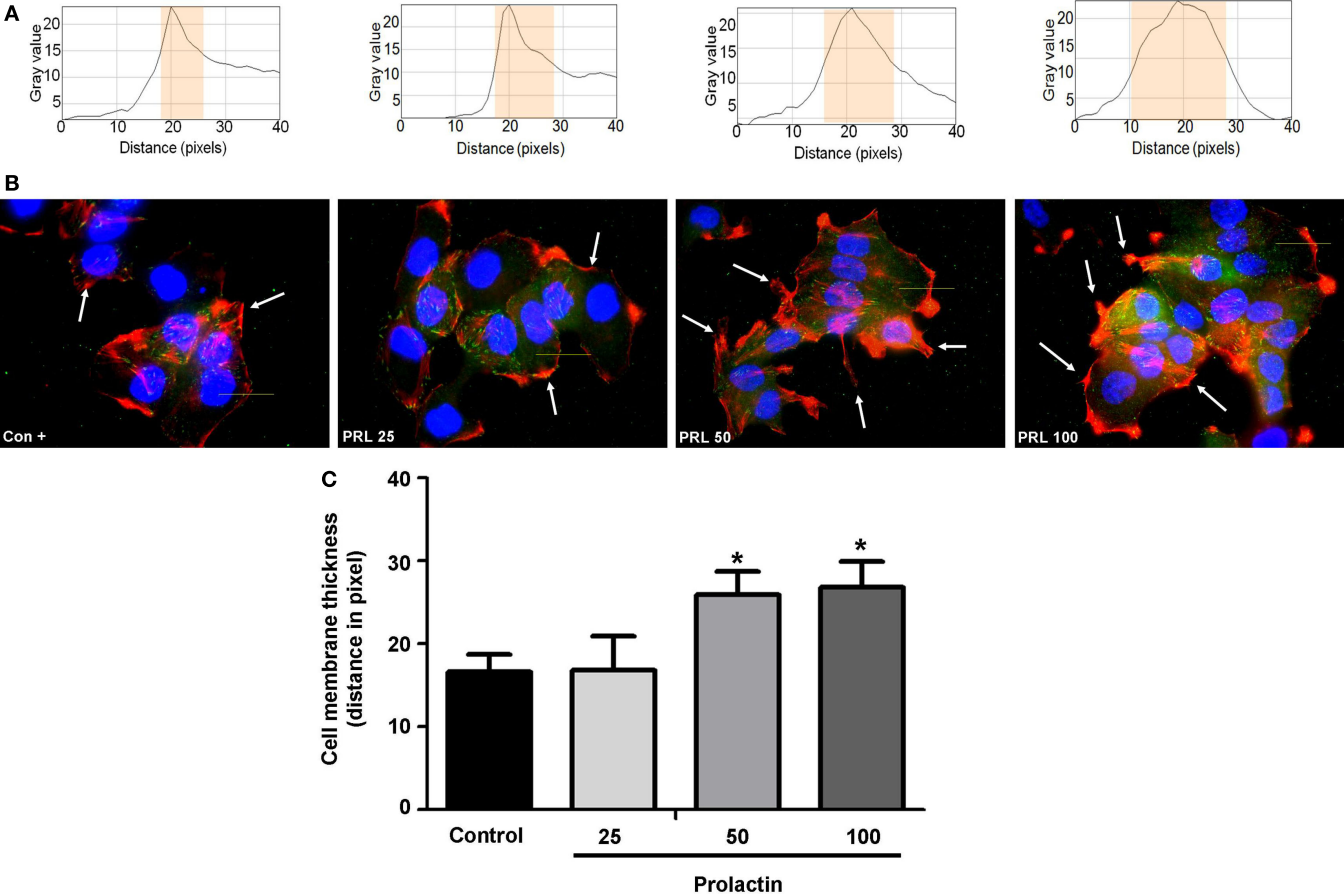
**(A)** The box on top of the cells display sample areas of measurement (one per cell, indicated as the yellow area). **(B)** shows T47D cellular actin stained with phalloidin linked to Texas Red (red staining) upon administration of different PRL concentrations (nanogram per milliliter). Nuclei were counterstained with DAPI (blue staining). **(C)** shows the mean thickness of the plasma membrane. Measurements were made in 50 different cells per condition with 5 different membrane thickness measurements per each cell. The experiment was carried out three times. *p < 0.05 in vs. control.

Cell movement is initiated by a global remodeling of the actin cytoskeleton. Actin redistribution toward the plasma membrane allows formation of membrane ruffles and pseudopodia where adhesion to extracellular proteins favors movement. We stained with Texas red-linked phalloidin the distribution of actin fibers and measured the thickness of plasma membrane area with immunofluorescence (Figure 2A), so to quantitatively assess actin membrane redistribution. We found that cells treated with PRL (at the concentration of 50 and 100 ng/ml) develop a rapid remodeling of actin toward the membrane, which corresponds to the formation of ruffles and pseudopodia (Figure 2B). Membrane actin accumulation during exposure to PRL is also shown by the statistically significant increase in thickness of the perimembrane area (Figure 2C).

### PRL Signaling Controlling Actin Remodeling

**Figure 3:**
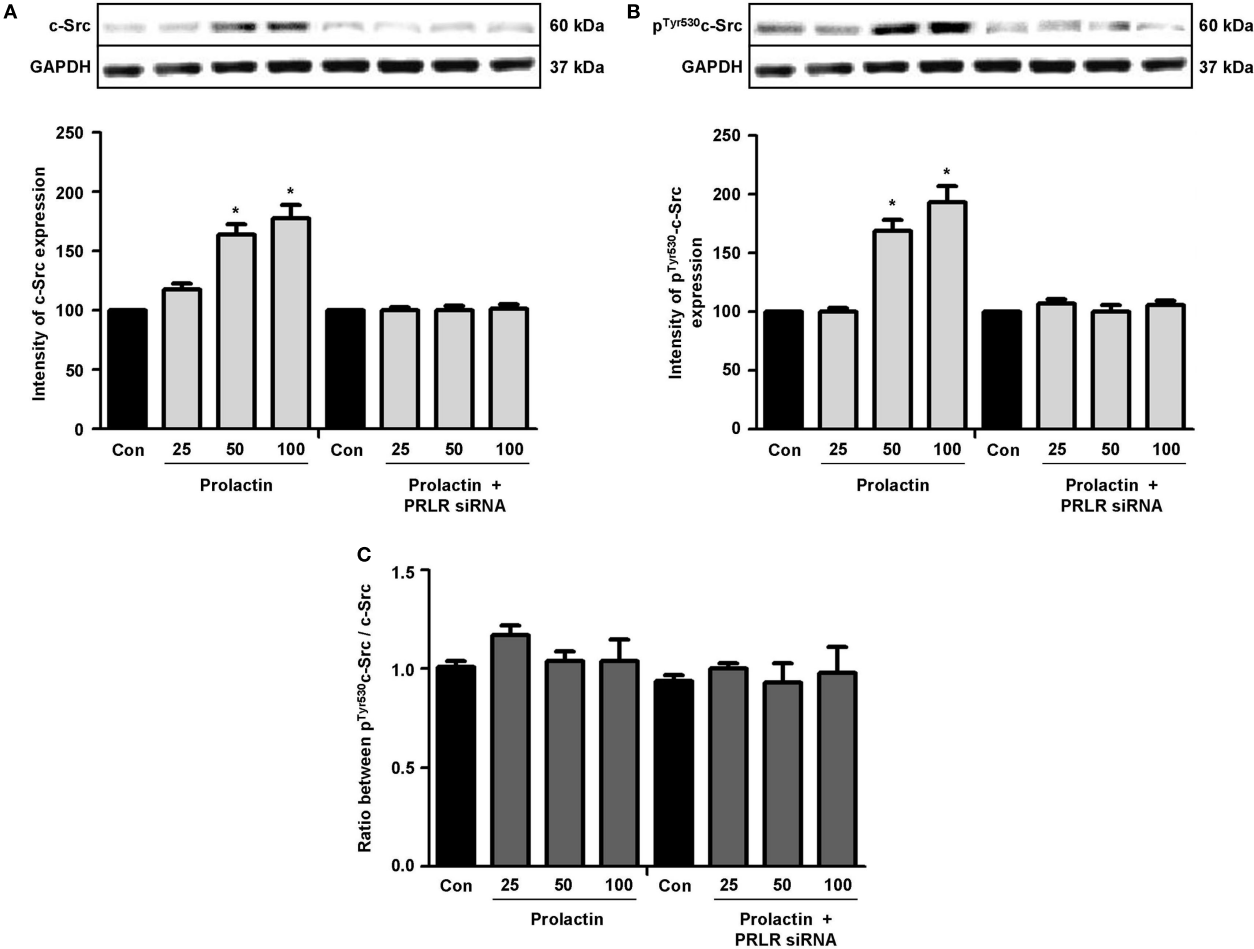
**The figure shows cellular amounts of c-Src (A) and p-^Tyr530^c-Src (B) upon administration of different PRL doses (nanogram per milliliter) in the presence or absence of anti-hPRLR siRNA**. The boxes show densitometric analysis of the WB bands adjusted for GAPDH and then normalized to control. **(C)** shows the ratio between c-Src and p-^Tyr530^c-Src. *p < 0.05 versus control. Experiments were repeated four times.

**Figure 4:**
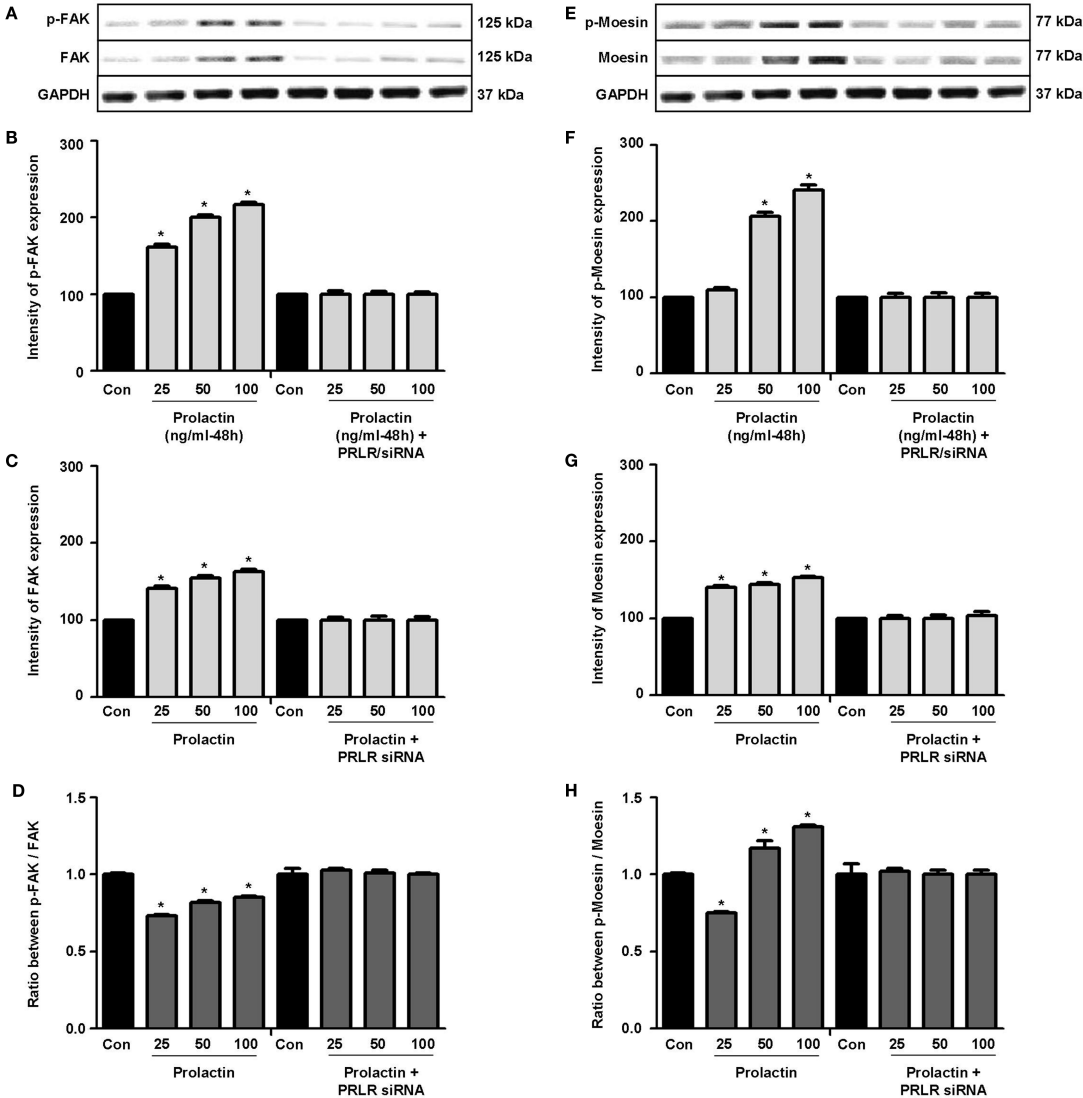
**(A,E)** show cellular amounts of P^Tyr397^FAK, FAK, p^Thr558^moesin, and moesin upon administration of different PRL doses (nanogram per milliliter) in the presence or absence of anti-hPRLR siRNA. **(B,C,F,G)** show densitometric analysis of the WB bands adjusted for actin and then normalized to control. **(D)** shows the ratio between FAK and P^Tyr397^FAK. **(H)** shows the ratio between moesin and p^Thr558^moesin. *p < 0.05 versus control. Experiments were repeated four times.

Actin remodeling is tightly controlled. Actin-binding proteins such as the Ezrin–Radixin–Moesin (ERM) protein moesin are requested to achieve fibrillar actin de-polymerization and relocation at the cell membrane (18). FAK is later required to form actin/vinculin-enriched structures called focal adhesion complexes, which mediate firm adhesion to the extracellular matrix (ECM) (10). Both these controllers are regulated by c-Src, which forms multiprotein signaling complexes with a number of membrane receptors. The cells exposed to PRL displayed elevated c-Src expression and a parallel increase in p-^Tyr530^ c-Src phosphorylation (Figures 3A–B) The Figure 3C shows the ratio between c-Src and p-^Tyr530^ c-Src. In parallel, increased expression of moesin and FAK, and enhanced ^Tyr397^FAK and ^Thr558^moesin phosphorylation were found in the cells (Figures 4A–H). Modulation of c-Src, moesin, and FAK expression and phosphorylation were abolished when PRLR was silenced with siRNAs (Figures 3 and 4).

## Discussion

The main result of this work is that PRL increases T47D, ZR75-1, and MCF-7 breast cancer cell movement by inducing structural changes in the cytoskeleton. This is achieved through signaling to the cytoskeletal controllers, moesin and FAK, and turns into a remodeling of actin position toward the cell membrane.

Previous evidence indicates that breast cancer cell motility is related to the abundance of PRL and PRLR transcripts (19–21), therefore suggesting the hypothesis that PRL signaling may have a role in this phenomenon. Understanding the means by which PRL–PRLR controls the interaction between cancer cells and the extracellular environment thereby influencing breast cancer cell motility may have clinical implications (22).

Several levels of evidence suggest that PRL may be directly associated with effects on ECM in the context of breast cancer. For instance, in a 3D analysis of collagen type 1, it was observed that the cells responded with increased ECM’s density after PRL stimulation (23). In addition, breast cancer progresses with the deposit of fibrillar collagen in the connective tissue near the tumor (desmoplasia) (24). In this context, PRL may induce reorganization of collagen, increasing the incidence of radially oriented fibers and modifying the stiffness of the tissue (25). This increased mechanical stiffness of the ECM is known to activate FAK and Src family kinases (SFK), thereby stimulating cellular motility (26). In breast cancer cells, SFKs triggered by PRL may generate pro-tumorigenic signals and stimulate cell motility. Although JAK2/STAT5 represents the principal physiological pathway of PRLR’s action, the signaling to SFKs by this receptor could also play a role in breast cancer progression (27).

It is peculiar that modulation of ECM by PRL seems to be specifically associated to progression of ER+ breast cancers. In this cancer subset, the extent and the type of geometric alignment of collagen fibers are predictive of biological aggressiveness (28). Approximately 75% of breast cancers express ERs. However, ER-targeted therapies fail in about 25% of cases (29). Changes in the ECM may thus represent a further area of investigation (23).

Prolactin receptor activation of Src family members has previously been studied in other cell types, such as hepatocytes, rat lymphocytes (Nb2 and W53), and even in mouse mammary epithelial cells (HC11) (10, 30). C-Src is activated through direct interaction with cell-membrane receptors, leading to auto-phosphorylation and therefore downstream signaling (31). Between the many protein kinases controlled by c-Src in breast cancer cells, the ERM family member moesin is a key mediator of cytoskeletal and cell membrane remodeling. When phosphorylated, activated moesin depolymerizes and remodels actin toward the plasma membrane, inducing the development of a cortical actin complex (32). Formation of this membrane actin platform is needed to develop transmembrane interactions between the cell’s cytoskeleton and proteins of the ECM. A second step that is relevant to achieve cell movement is the formation of focal adhesion complexes, or structures enriched in actin and vinculin where bridges between the cortical actin complex and the ECM are formed through anchoring proteins of the integrin family (33). This process is mediated by FAK that promotes the development and the turnover of adhesion complexes (34). Later contraction of the cytoskeleton allows cell movement. Moesin and FAK are overexpressed in breast cancer, and their level of expression is related to the metastatic potential (35, 36).

Plasma levels of PRL seem to represent a risk factor for breast cancer metastasis (37). Overexpression and functional activation of FAK, moesin, and c-SRC in breast cancer cells may in part explain this epidemiologic finding. Based on our results, it may be hypothesized that some of the effects of PRL on breast cancer cell motility may be triggered through recruitment of the small adapter protein, c-Src, and to FAK and moesin recruitment, followed by actin rearrangement and enhancement of cell motility.

In summary, we here show that T47D, ZR75-1, and MCF-7 cell motility is enhanced by exposure to PRL. This leads to PRLR-dependent signaling to c-Src, moesin, and FAK, and to actin cytoskeleton remodeling that is related to cell motility. These finding highlight new signaling avenues through which PRL may influence the biological behavior of breast cancer. These pathways may be investigated as new targets for intervention to decrease the ability of breast cancer progression.

## Author Contributions

PS and VA carried out the majority of the experiments ­contributed to the conception and design of the work and drafted the manuscript, VG and MG performed immunofluorescence assays, PM performed migration assays, EB and JS participated to the writing and revision of the manuscript and elaborated the results, TS planned and supervised the experiments and wrote the paper.

## Conflict of Interest Statement

The authors declare that this research was conducted in the absence of any commercial or financial relationships that could be construed as a potential conflict of interest.
